# Efficient pneumonia detection using Vision Transformers on chest X-rays

**DOI:** 10.1038/s41598-024-52703-2

**Published:** 2024-01-30

**Authors:** Sukhendra Singh, Manoj Kumar, Abhay Kumar, Birendra Kumar Verma, Kumar Abhishek, Shitharth Selvarajan

**Affiliations:** 1 JSS Academy of Technical Education, Noida, India; 2 National Institute of Technology Patna, Patna, India; 3 School of Built Environment, Engineering and Computing, Leeds Beckett University, LS1 3HE, Leeds, UK

**Keywords:** Computational science, Computer science, Information technology

## Abstract

Pneumonia is a widespread and acute respiratory infection that impacts people of all ages. Early detection and treatment of pneumonia are essential for avoiding complications and enhancing clinical results. We can reduce mortality, improve healthcare efficiency, and contribute to the global battle against a disease that has plagued humanity for centuries by devising and deploying effective detection methods. Detecting pneumonia is not only a medical necessity but also a humanitarian imperative and a technological frontier. Chest X-rays are a frequently used imaging modality for diagnosing pneumonia. This paper examines in detail a cutting-edge method for detecting pneumonia implemented on the Vision Transformer (ViT) architecture on a public dataset of chest X-rays available on Kaggle. To acquire global context and spatial relationships from chest X-ray images, the proposed framework deploys the ViT model, which integrates self-attention mechanisms and transformer architecture. According to our experimentation with the proposed Vision Transformer-based framework, it achieves a higher accuracy of 97.61%, sensitivity of 95%, and specificity of 98% in detecting pneumonia from chest X-rays. The ViT model is preferable for capturing global context, comprehending spatial relationships, and processing images that have different resolutions. The framework establishes its efficacy as a robust pneumonia detection solution by surpassing convolutional neural network (CNN) based architectures.

## Introduction

Pneumonia is a common respiratory infection caused by multiple types of bacteria, viruses, and fungi. It is the leading cause of morbidity and mortality worldwide, particularly among infants under the age of five and the elderly. According to WHO^1^, 1.4 million pneumonia-related fatalities among children under five in 2018. Chest X-ray imaging is commonly used to diagnose pneumonia, as it can reveal important symptoms, such as increased lung opacity and consolidation. However, it can be difficult to interpret a chest X-ray (CXR) because pneumonia symptoms can be subtle and overlap with other lung diseases. Rapid and accurate diagnosis of pneumonia is essential for expediting treatment and improving patient outcomes. Radiological images, such as chest X-rays or CT scans, require specialized training and can be time-consuming to diagnose pneumonia.In recent years, there has been significant interest to develop model using machine learning techniques that assist physicians in diagnosing pneumonia using chest X-ray images. These techniques have shown promising results and may improve the efficacy and accuracy of pneumonia diagnosis.

**Figure 1:**
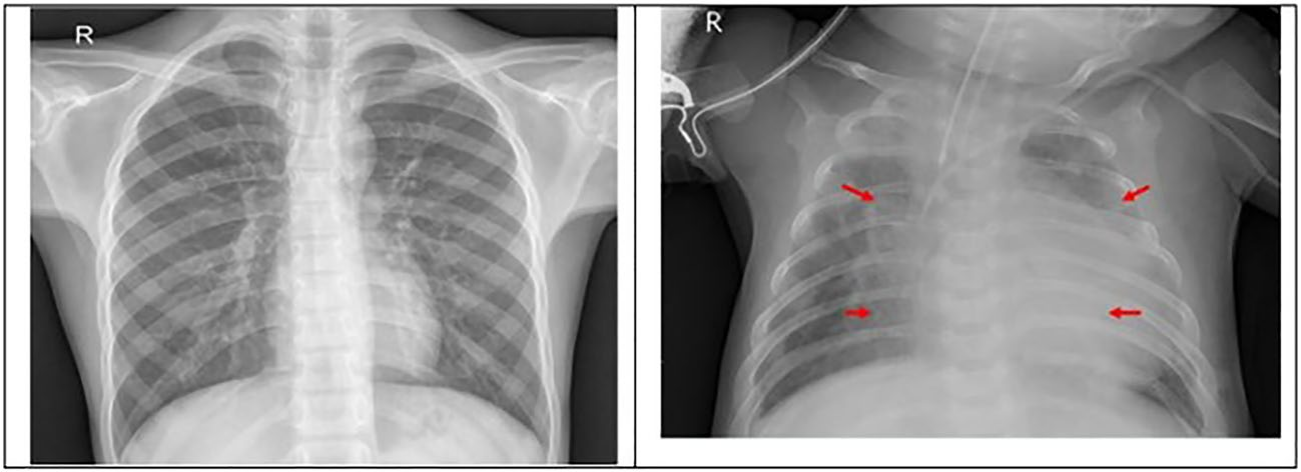
A sample CXR (normal and pneumonia) image.

By training a CNN on a dataset of chest X-ray images, Deep Learning (DL)^2–5^ has been utilized to detect pneumonia^6–10^. As shown in Fig. 1, the CNN can learn to recognize patterns and associated features with pneumonia, such as clouded lung areas to detect pneumonia.The model can then be used to classify new X-ray images as normal or pneumonia. Multiple studies^11–14^ have demonstrated the efficacy of this method in detecting pneumonia with a high degree of accuracy. Attention mechanism isn DL refers^15–21^ to a technique used in neural networks to selectively focus on certain portions of an input as opposed to processing the entire input equally. In image detection and classification, attention mechanisms can be utilized to concentrate the network's attention on specific regions of an image that are most important for making a classification decision. This can help the network to improve its accuracy and decrease its computation needs. ViT models are a variant of the Transformer architecture^22–26^, which was originally designed for NLP applications. These models have been adapted for image classification tasks by handling an image as a sequence of image segments that are then processed by the transformer's attention mechanism. In addition, the ViT model outperformed state-of-the-art (SOTA) techniques on a broad variety of image classification tasks, making it an excellent candidate for the pneumonia diagnosis task.

### Motivation

Vision Transformer architecture for pneumonia detection from CXR is motivated by the need for time to detect this severe respiratory disease. Globally, pneumonia is one of the big causes of mortality. Early diagnosis and treatment are crucial for improved patient outcomes. Traditional methods of evaluating CXR to diagnose pneumonia are time-consuming and require specialized medical knowledge, which can lead to diagnostic errors and treatment delays. In response to these challenges, DL techniques such as CNNs and RNNs have been developed to automate the detection of pneumonia from CXR. However, these methods are inadequate to analyze complex medical images. ViT architecture has demonstrated exceptional efficacy in a variety of vision tasks, including image classification and object detection. It is a viable candidate for pneumonia detection from CXR because it can extract global and local image features. Utilizing the power of self-attention mechanisms, ViT is able to effectively capture complex patterns and relationships in X-ray images, resulting in improved pneumonia detection accuracy and reliability. Therefore, the goal of utilizing ViT architecture for pneumonia detection from CXR is to surmount the limitations of conventional methods and improve the precision and efficacy of DL models for medical imaging analysis. Vision Transformer architectures are totally different from CNN architectures. Transformer-based architectures were initially designed for sequence-to-sequence tasks in natural language processing. CNN is primarily used for tasks like machine translation, text summarization, language modeling, and sentiment analysis. These architectures have been customized into Vision Transformer architecture so that they can be suitable for Image classification and analysis.

The contribution of work is summarized as follows.

- In this investigation, we propose a ViT-based architecture for pneumonia detection in CXR. This architecture will be designed to effectively manage the large and complex medical images that are typical in CXR and will be capable of detecting pneumonia with precision.
- We will evaluate the accuracy of the proposed ViT architecture to that of existing DL techniques. This will provide a thorough analysis of the benefits and drawbacks of our proposed approach compared to existing methodologies.
- We will evaluate the efficacy of the proposed ViT architecture using a CXR dataset that is publicly available. This will entail training and testing the model using a set of performance metrics, including accuracy, recall, precision, and F1 score, to measure its performance.

We will present the proposed ViT architecture's performance evaluation findings and analysis. This will include a discussion of any limitations of the proposed model and recommendations for improving its efficacy through future work.

#### Organization of the paper

The rest paper is structured as Sect. 2 discusses the background and working principle of the proposed architecture and other variants of Vision Transformer architecture. Section 3 presents recent applications and a review of related studies. Section 4 describes the dataset characteristics and proposed architecture. Section 5 discusses experiment specifications, results, and prospects of Vision Transformer architecture, followed by Sect. 6, which represents the conclusion.

## Background and methodology

In this section, the paper builds the foundation for the proposed architecture.

### Transformer architecture

The transformer architecture is a neural network^27^ designed for natural languages, such as language translation, language modeling, and text summarization. The main concept of the transformer architecture is the self-attention mechanism, which assess the relative relevance of various words or sub-phrases in a given input. This is achieved by computing a "query," "key," and "value" for each word or sub-phrase, followed by adding a weighted sum on the similarity between the query and the keys. Additionally, the transformer architecture utilizes a multi-head attention mechanism^28–30^ to attend to various input positions. In addition to the self-attention mechanism^31^, the feed-forward neural network process the output of the self-attention layer to produce the better result in Transformer model. The architecture also uses positional encoding to convey the position of the input image.

### Vision transformer derived from generic transformer architecture

The Vision Transformer replaces the original transformer's self-attention mechanism with a spatial attention mechanism^32^ which is designed to govern images' two-dimensional grid structure. This enables the model to analyze and comprehend the spatial relationships between different image regions. Itis an effective architecture for image classification and computer vision tasks. Images are processed through the Transformer model, consisting of spatial attention and a feed-forward neural network. The spatial attention mechanism applies the attention to the image pixels, followed by the feed-forward neural network to the output of the attention mechanism. In addition, this modeluses a patch-based strategy where an image is divided into smaller segments and learns to focus separately on each patch. This allows the model to extract granular features and improve its accuracy.

#### Working principle of Vision Transformer

The fundamental concept of a ViT is the self-attention mechanism, which exploits both global and local features by focusing on distinct portions of the image. The self-attention mechanism is implemented by adding self-attention layers with multiple heads that are known as transformer blocks. Each patch is converted into corresponding 1-D vector and transmitted to the transformer. The transformer then uses self-attention to learn the relationships between the various regions, and the resulting representation is input into a feed-forward neural network to make a prediction.As the spatial resolution of the input does not constrain the self-attention mechanism, one of the main advantages of ViT is their ability to handle images of arbitrary sizes. This model can be trained on large images, such as high-resolution medical images, without downsampling or cropping. Additionally, this model has been improved in recent variants such as DeiT^33,34^, Swin-T^35,36^, and ReViT^37^ to enhance their performance, reduce the number of parameters and computational costs, and make them more efficient and scalable for practical applications.

### Self-attention mechanism in Vision Transformer for image detection and classification

A Vision Transformer^38,39^ is a neural network that processes visual information using self-attention mechanisms. Similar to how the Transformer architecture is used in natural language processing (NLP), ViT employs attention mechanisms to evaluate the specific parts of an image in order to make accurate predictions. These networks excel at image classification and object detection.

#### Self attention techniques

Self-attention^15^ is a technique that enables a model to selectively concentrate its processing on particular regions of an image. Self-attention is typically applied to extracted feature maps generated by a CNN in the context of images. Self-attention allows the model to determine the relative importance of various image regions by computing a set of attention weights for each region. These attention weights can then be applied to the feature maps before their transmission to the remainder of the network.There are numerous methods to incorporate self-attention into images. A common technique is using a multi-head self-attention mechanism, in which the model computes multiple sets of attention weights for various regions of the image and then combines them. This allows the model to consider the entire image when making a prediction rather than just a specific region's features. A further method for image processing is to use a transformer-based model in which the self-attention mechanism focuses on various image regions when selecting a prediction. The transformer-based model is trained to understand the relationships between multiple image regions and makes predictions based on this information.

Self-attention in DL for image processing can be categorized into two main modules: channel attention and spatial attention.

##### Spatial attention networks

In contrast to conventional CNNs, which process entire images and extract features from them, spatial attention networks^32,40^ process only particular regions of an image. This is accomplished by incorporating an attention mechanism that learns to weigh various image regions based on their significance to the current task. By selectively attending to the relevant areas of an image, spatial attention networks can achieve greater accuracy and efficiency when performing tasks such as image captioning, object detection, and visual question answering. In addition, the attention mechanism improves the interpretability of these networks by highlighting the regions of the image that the network is concentrating on for a given task.

##### Channel attention

Channel attention^41,42^ pertains to a mechanism's ability to focus on particular channels of the feature maps selectively. Typically, this is carried out by computing a set of attention weights for each channel of the feature maps. These attention weights can then be applied to channels before their transmission through the remainder of the network. This allows the model to concentrate its prediction on the channels that are most informative.The combination of channel and spatial attention empowers the model to predict using both spatial information (the location of the specified portion within the image) and channel information (the features extracted by the CNN). This results in more robust and generalizable modelsfor images that have not been seen.

#### Variants of Vision Transformer

Several customizations in ViThave been experimented with to improve its performance or fit certain applications. The main customization methods include.

##### Patch size

The ViT architecture linearly embeds fixed-size input image patches. Patch size affects model performance. Larger patches capture global context but lose fine-grained details, while smaller patches may fail to capture global context. To find better performance, optimal patch size has been used.

##### Positional encoding

ViT incorporates spatial information into the model via learnable positional encodings. These encodings assist the model understand image patch placements. ViT performance can be improved with sine/cosine, spatial, or learned positional encodings.

##### Architectural variations

To improve ViT, researchers have tried several architectural variations. A Pyramid Vision Transformer (PVT) is a hierarchical modification that captures multi-scale information. The Convoluted Vision Transformer (ConvViT) combines self-attention and convolutional layers to use local and global information.

##### Training methods

ViT performance and convergence have been improved using various training methods. Data augmentation, regularization (dropout, weight decay), and advanced optimization algorithms (Adam, RMSprop) are examples. Pretraining on ImageNet and transfer learning^43,44^ have also been used to initialize ViT models.

##### Hybrid models

Hybrid designs integrate Convolutional Neural Networks (CNNs) and Vision Transformers (ViTs) for tasks such as pneumonia detection in chest X-ray images, we first use a CNN as the feature extractor, removing its fully connected layers while retaining its convolutional and pooling layers. The CNN-generated feature maps are then separated into non-overlapping patches, and each patch is converted into a high-dimensional embedding vector. These embeddings, which depict local characteristics, are then fed into the ViT model in order to capture global dependencies and contextual information across the entire image. For final predictions, a classification head is appended to the ViT output. The entire hybrid model, comprised of the CNN feature extractor and the ViT model, is trained from beginning to end using labeled data, with fine-tuning strategies tailored to the specific dataset and computational resources available. This approach maximizes the extraction of both local and global information, optimizing performance for complex image analysis tasks.Transformers process CNN-extracted features. This hybrid strategy uses CNNs (local feature extraction) and transformers (global context modeling) to improve performance. Pyramid Vision Transformer (PVT captures multi-scale information hierarchically. Multiple steps process features at varying resolutions. The model effectively captures local and global information. A convoluted Vision Transformer (ConvViT) is a Self-attention mechanism with convolutional layers. Self-attention models global context, while convolutional layers catch local patterns. This combination improves the model's local and global information handling.

##### Attention mechanism

ViT's architecture relies on attention techniques. Attention mechanism customization may include Long-Range Arena (LRA) attention, Axial attention, and Shifted attention. LRA attention efficiently handles input image long-range dependencies. It helps the model capture global context even when patches are far apart.

Axial attention captures dependencies along image axes (rows and columns). Self-attention is modified to catch shifted or offset patch dependencies. This helps the model manage data spatial transformations.

To have state-of-the-art performance and improved convergence,researchers have experimented with the following pre-trained Vision Transformer architectures.

##### DeiT (data-efficient image transformers)

DeiT^34^ uses self-attention mechanisms and patch-based processing to outperform CNNs in image tasks with less labeled training data. Self-attention computes attention weights on smaller image patches to efficiently capture long-range relationships and grasp the global context. The models are pre-trained on large, unlabeled datasets to learn general visual representations, then fine-tuned on smaller, task-specific datasets. Visual characteristics and hierarchical representations help the model transfer pre-trained knowledge to the target task. Dropout and data augmentation increase generalization. Data-efficient image transformers use self-attention, patch-based processing, pre-training, fine-tuning, transfer learning, and regularization to perform well in picture tasks without labeled data.

##### Swin-T

Swin transformer^36,45^, a new image understanding architecture, blends Transformers with CNNs. It converts the input image into non-overlapping patches using transformer layers. Swin Transformer's hierarchical architecture organizes transformer layers into stages, making it unique. Lower stages process patch-level information, whereas later stages capture broader contextual information. The hierarchical model efficiently captures image local and global dependencies. Shift procedures help Swin Transformer model repair spatial links. Swin Transformer uses Transformers' self-attention mechanism and CNNs' efficient processing to achieve state-of-the-art results on image classification, object detection, and semantic segmentation with fewer computational resources than other transformer-based models.

##### ReViT

The Vision Transformer (ViT) architecture can accommodate inputs of different resolutions with Resizable-ViT^37^. Traditional ViT models require fixed-size inputs, which can limit their adaptability in real-world applications with varied image sizes. Resizable-ViT solves this problem with "token shifting" and "layer dropping." Token shifting requires scaling the input image and adapting position and token embeddings to the new resolution. For lower inputs, layer-dropping skips model architectural layers based on input resolution, reducing computing complexity. Resizable-ViT efficiently processes images of varied resolutions while doing well on image recognition tasks by dynamically adapting to input sizes.

All of these variants have been shown to enhance the performance and efficiency of Vision Transformers and have been applied to a variety of tasks, including image recognition, object detection, and medical imaging, with SOTA results.

## Recent applications of Vision Transformer architecture

Vision Transformer (ViT) has attracted great interest in computer vision duties due to its capacity to process images with high precision and efficiency. Recent developments and applications have been made to the ViT architecture. The DeiT model, which enhances the training of ViT models using data augmentation and distillation techniques, is one of the most significant innovations. The Swin Transformer model, which employs hierarchical representations to enhance the performance of ViT models on large-scale image datasets, is another innovation.Recent Vision Transformer architectures research has centered on a variety of applications, including.

### Object detection and instance segmentation

ViT architecture is promising for object detection and instance segmentation because it possesses several essential characteristics that make it suitable for these tasks. First, the self-attention mechanisms in ViT enable the model to learn global relationships between various image components, which can be used to identify and localize objects. ViT can be trained on large datasets with many labeled examples, which is essential for these tasks because they require a large amount of data to learn the involved complex patterns. Finally, ViT can be fine-tuned for specific object detection or instance segmentation tasks^46^, allowing it to achieve high accuracy by adapting to the requirements of these tasks.

### Dense predictions

Dense prediction is the task of predicting a pixel-wise output for an input image, such as semantic segmentation, where each pixel is designated as a specific object or background. The input image is divided into a series of non-overlapping segments for dense prediction, which is then flattened and fed into the ViT architecture. Self-attention allows ViT to record spatial information across these regions, and the output is shaped into a grid corresponding to the original image. One of the benefits of employing ViT for dense prediction is that it can learn to distinguish between objects of varying sizes and shapes without explicit object proposals or region-based attributes. ViT attends to all regions in the input image and learns to weigh their contributions based on the significance of their contributions to the output. In addition, ViT can be trained end-to-end with large-scale datasets like ImageNet to acquire general features that can be applied to subsequent tasks like a dense prediction. In situations with limited labeled data, this makes ViT an attractive design for dense prediction.

### Self-supervised learning

Even without human annotations, ViT can be used for self-supervised learning^47,48^. Self-supervised learning teaches input data meaningful representations for classification, detection, and segmentation. Training the model on a pretext task is one method to use ViT for self-supervised learning^49^. Pretext tasks allow the model to learn key characteristics from input data. Data augmentation to generate multiple perspectives of the same image and training the ViT model to predict which views match is a common pretext challenge. Contrastive learning teaches the ViT model to distinguish between similar and distinct images. Two arbitrary images are supplied to the ViT model. The model is then trained to predict whether or not two images are identical.In both cases, the ViT model discovers features that are independent of viewpoint, illumination, and other factors that affect the appearance of input data. These learned characteristics can be used to establish supervised model weights or to refine subsequent tasks.

### Multi-modal learning

Recent research^50^ has examined the use of transformer-based architectures for multimodal unsupervised learning from raw video, audio, and text. Using self-supervised learning techniques, the plan is to implement a transformer-based architecture capable of handling multiple modalities and capable of predicting the next frame, audio, or text given the current one.

### Efficient ViT architectures

Recent efforts have been made to make Vision Transformer models more effective in terms of computation time and memory consumption. Multiple architectures, such as Separable Vision Transformer (SepViT)^51^ and Reversible Vision Transformer (RViT)^37,52^, have been proposed by researchers that are capable of achieving comparable or superior performance than conventional ViT models while being more energy-efficient. SepViT blocks employ separable convolutions rather than conventional convolutions. This update minimizes the self-attention mechanism, the most computationally expensive component of ViT. Separable convolutions separate conventional convolutions into depthwise and pointwise convolutions, requiring fewer parameters and computations. RViT augments ViT design with reversible residual blocks. These blocks recreate input features from output features, which increases the efficiency of gradient calculation during backpropagation. Reversible blocks enable models with limited memory to be larger.

### Explainable AI

ViT can be utilized in Explainable AI^33^ to provide insight into how an image classification decision is made. By using attention maps generated by ViT, it is possible to visualize which aspects of an image are most crucial to the classification decision. This information can be used to clarify the model's decision when communicating with humans.

In Table 1, the article summarizes recent contributions made for a range of tasks using Vision Transformer architecture.

**Table 1:** Insight into related recent research.

Article references	Approach	Major findings	Gap identified and future direction for enhancement
“An Image is Worth 16 × 16 Words: Transformers for Image Recognition at Scale”^53^	Each segmented patch is linearly projected into a high-dimensional embedding space that result is then input into the Transformer encoder.They replaced the traditional CNN backbone with a Transformer encoder-decoder framework, thereby enabling a more unified framework across modalities obtained cutting-edge performance on benchmark datasets with fewer computational resources than traditional CNN-based methods	results can be improved by adjusting the number of layers, the dimensionality of the embeddings, or the design of the attention mechanism and by fine-tuning the architecture to strike a balance between model capacity and computational efficiency	Transformers demand more processing power and memory than convolutional neural networks (CNNs), and the article does not elucidate how to address this. Transformers are less interpretable than CNNs, and interpretability strategies are not discussed in the article. Patch size, computational efficiency, and performance compromises are not considered. Resolving these issues could facilitate the scalability of image recognition methods based on the Transformer
“Show, attend and tell: Neural image caption generation with visual attention”^17^	The authors demonstrate the effectiveness of incorporating a visual attention mechanism into the caption generation process. The attention mechanism allows the model to focus on various portions of the image while generating each word in the caption, thereby improving the alignment between the image content and the generated text	superior caption quality in comparison to previous methods. By focusing on pertinent image regions, the model generates more accurate and descriptive captions that capture the image's most important objects, actions, and relationships	The approach lacks fine-grained attention because it employs a mechanism for soft attention that assigns weights to image regions rather than concentrating on particular objects or attributes. This hinders the capability of the model to generate captions with precise details. The article does not discuss strategies or techniques for fine-tuning the interpretation and control of the attention mechanism, thereby limiting the adaptability and interpretability
“Deep MRI Reconstruction with Generative Vision Transformers”^54^	Deep generative network GVTrans translates noisy variables and latent onto high-quality MR images. Multi-layer architecture improves image resolution. Cross-attention transformer modules receive up-sampled feature maps in each layer. MR images are masked using the same sampling pattern as the under-sampled acquisition for test data inference. Optimized network parameters ensure that reconstructed and original k-space samples match	better image quality than CNN-based reconstructions with and without self-attention processes and can adjust to individual test subjects. GVTrans may improve deep MRI reconstruction applicability and generalizability	Using a larger dataset of fully-sampled MRI acquisitions for training GVTrans, incorporating additional information, such as patient demographics or clinical history, into the training process, and developing a more efficient training algorithm for GVTrans can improve the performance of the proposed architecture GVTrans.Training in the proposed GVTrans architecture is computationally intensive.GVTrans may be unable to reconstruct images with high levels of noise or anomalies, as well as images with very low sampling rates
“A Simple Single-Scale Vision Transformer for Object Detection and Instance Segmentation”^46^	Universal Vision Transformer (UViT), an intuitive and efficient Vision Transformer architecture, was proposed for object detection and instance segmentation	UViT is a simple yet efficient model that achieves competitive performance on the COCO benchmarks for object detection and instance segmentation	On some tasks, such as dense prediction, UViT may not attain the same level of performance as more complex Vision Transformer architectures. UViT may not be as effective as models for object detection and instance segmentation that are more specialized
“Training data-efficient image transformers & distillation through attention”^34^	A large, pre-trained convolutional neural network (CNN) is used as a teacher to train a smaller, more efficient transformer-based student model in this method. The student model gains knowledge from the teacher by observing the instructor's output, which is represented by a distillation token. The distillation token is added to the input of the student model and is utilized to direct the attention mechanism	DeiT-B model obtains 85.2% top-1 accuracy on ImageNet with 86 M-parameterwhen trained with 100 epochs and 16 GPUs	The distillation token can be computationally expensive to compute, which is a limitation. Another limitation is that the distillation token can result in a reduction in the attention weights' diversity.It would be possible to enhance the distillation token by employing a more efficient method for computing it. The distillation token could be modified to promote attention weights with greater diversity. The method could be applied to additional tasks, including object detection and segmentation
“Analyzing Transfer Learning of Vision Transformers for Interpreting Chest Radiography”^55^	utilizing a standard Vision Transformer architecture and training it on a large collection of natural images. Using a limited number of labeled examples, they then refined this model using the CheXpert or Pediatric Pneumonia dataset	A model's performance on a medical image classification task can be considerably enhanced by transfer learning from a previously trained Vision Transformer. There is no significant effect on the efficacy of the model by fine-tuning	Domain adaption and other transfer learning methods may improve Vision Transformers' medical image classification performance in future research. The model’s performance can further be improved using larger fine-tuning datasets
“Introducing Convolutions to Vision Transformers”^56^	a novel design called Convolutional Vision Transformer (CvT) that increases Vision Transformers (ViTs) performance and efficiency by adding convolutions.A convolutional token embedding layer replaces the token embedding layer. This enables the CVT to discover spatial relationships between tokens, thereby enhancing the model's capacity to represent complex visual patterns. Convolutional attention operation replaces the attention operation. This enables the CvT to efficiently compute attention weights across vast spatial regions, thereby enhancing the model's capacity to capture global context	CvT outperforms ViTs on a variety of image classification tasks while requiring fewer parameters and FLOPs. For instance, the CvT achieves a top-1 accuracy of 89.4% on the ImageNet-1 k dataset, which is comparable to the state-of-the-art performance of ResNet-50 despite employing only 1/10th of the parameters and 1/100th of the FLOPs	CvTs are harder to train and slower at inference hen compared with ViT's. Using deeper and broader CvT models to further improve performance, adding residual connections between CvT layers to improve training stability, and employing dilated convolutions and group convolutions to improve the model's ability to represent long-range dependencies can further improve the proposed model

## Material and methods

### Dataset characteristics

In the investigation, we used a publicly available chest X-ray (CXR) dataset from Kaggle^57,58^. The same dataset has also been utilized in numerous other investigations. The dataset consists of three sections: train, test, and validation. Each section contains subfolders for Pneumonia and Normal CXRs. There are 5863 X-ray images in total as shown in Table 2. The X-ray images used in the dataset were acquired at the Women and Children's Medical Center in Guangzhou from children aged one to five.These images were taken as part of the children's routine medical examinations.To assure the quality of the X-ray images used in the analysis, they were screened by specialists for low-resolution or unreadable images. The remaining images were then evaluated by two physician specialists, with any discrepancies resolved by a third specialist. This procedure was performed to teach an AI system to make precise diagnoses.80% of the dataset has been allocated to the training set, 10% to the test set, and 10% to the validation set, as shown in Table 3.

**Table 2:** Class distribution of the dataset.

Class	No of images
Pneumonia (P)	4273
Normal (N)	1583

**Table 3:** Partitioning of training, testing, and validation datasets.

	# of images	# of images from P class	# of images from N class
Training data	4684	3205	1479
Validation data	586	360	226
Test data	586	330	256

### Proposed architecture

**Figure 2:**
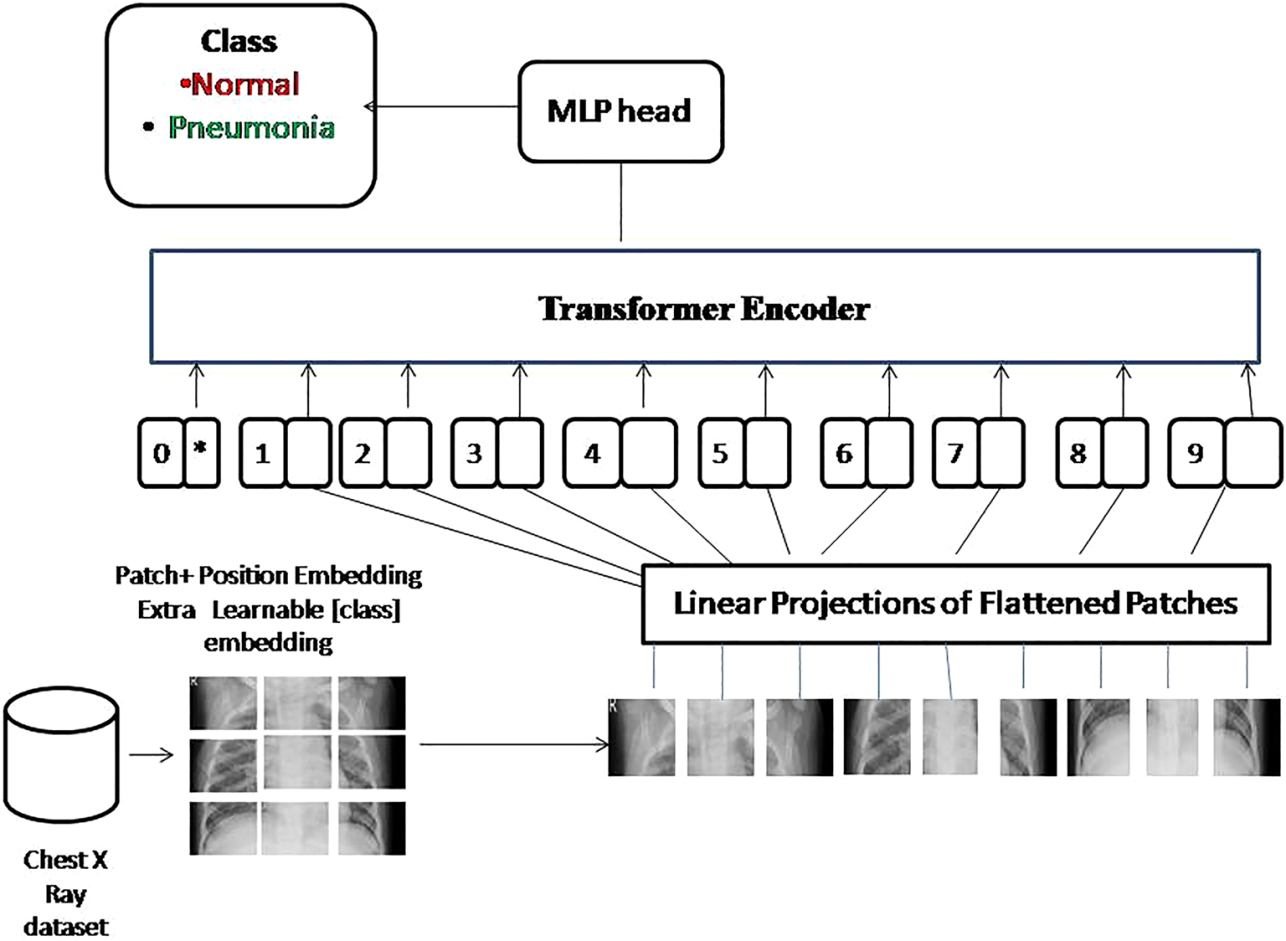
The proposed system design architecture.

The proposed Architecture uses patch embeddings, positional encodings, several Transformer encoder layers, self-attention, feed-forward neural networks, and a classification head to classify and analyze imageswhich are shown in Fig. 2.

#### Input embedding

**Figure 3:**
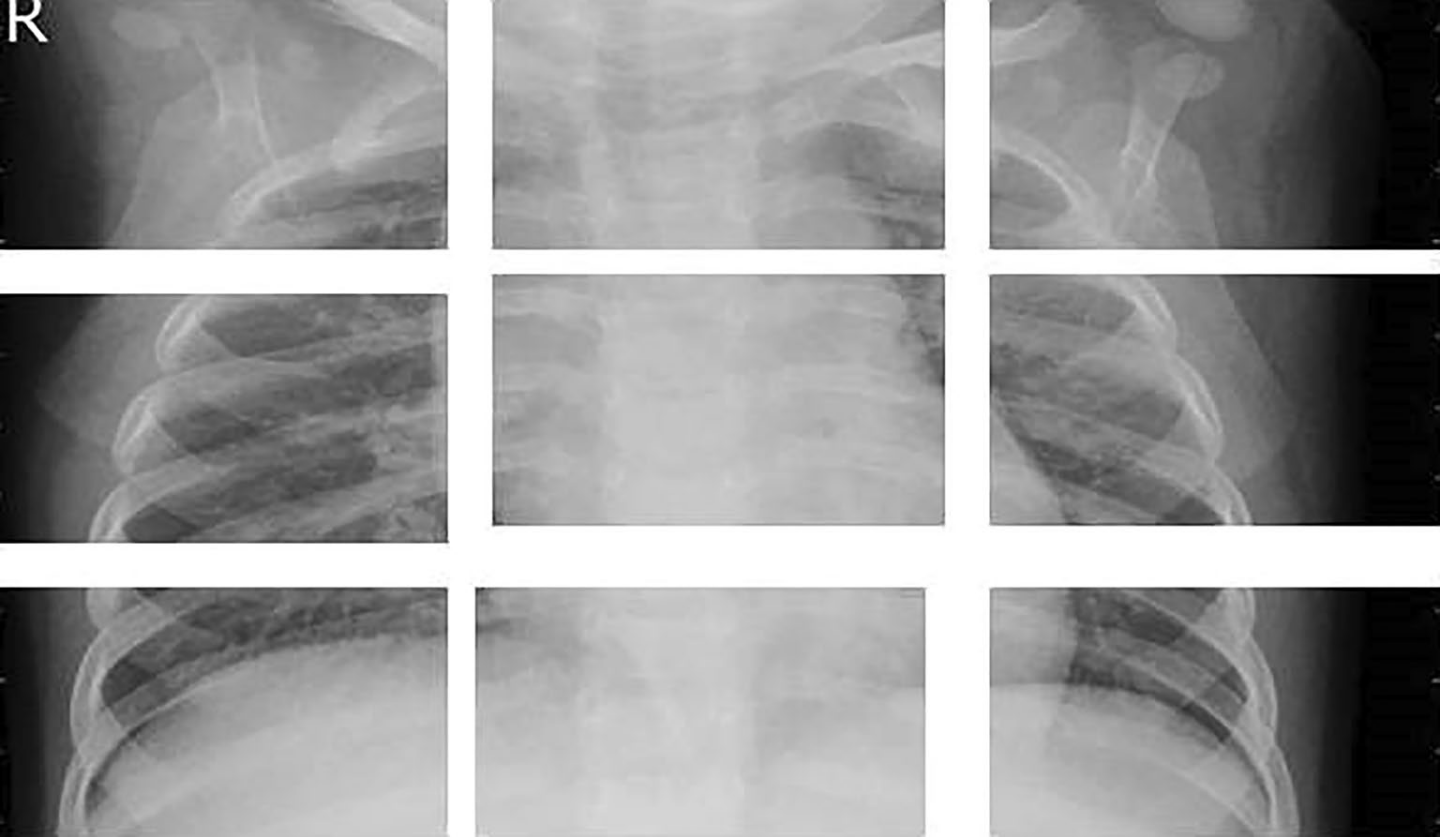
Dataset input image in the form of smaller patches.

It requires reshaping the input image into patches as shown in Fig. 3 and applying a linear transformation in order to obtain the embeddings. Let's denote the input image as $X\in R^{(H\times {\text{W}}\times {\text{C}})}$ re H, W, and C, respectively, represent the height, breadth, and number of channels. Each patch has a dimension of P × P, and there are N patches in total. The input embedding can be represented the as $E\in R^{(N\times {\text{D}})}$ where D is the number of dimensions of the embeddings.

#### Positional encoding

The input embeddings include positional information to capture the relative and absolute positions of the patches. The positional encoding matrix $P\in R^{(N\times {\text{D}})}$ added to the input embeddings E element by element.

#### Transformer encoder

**Figure 4:**
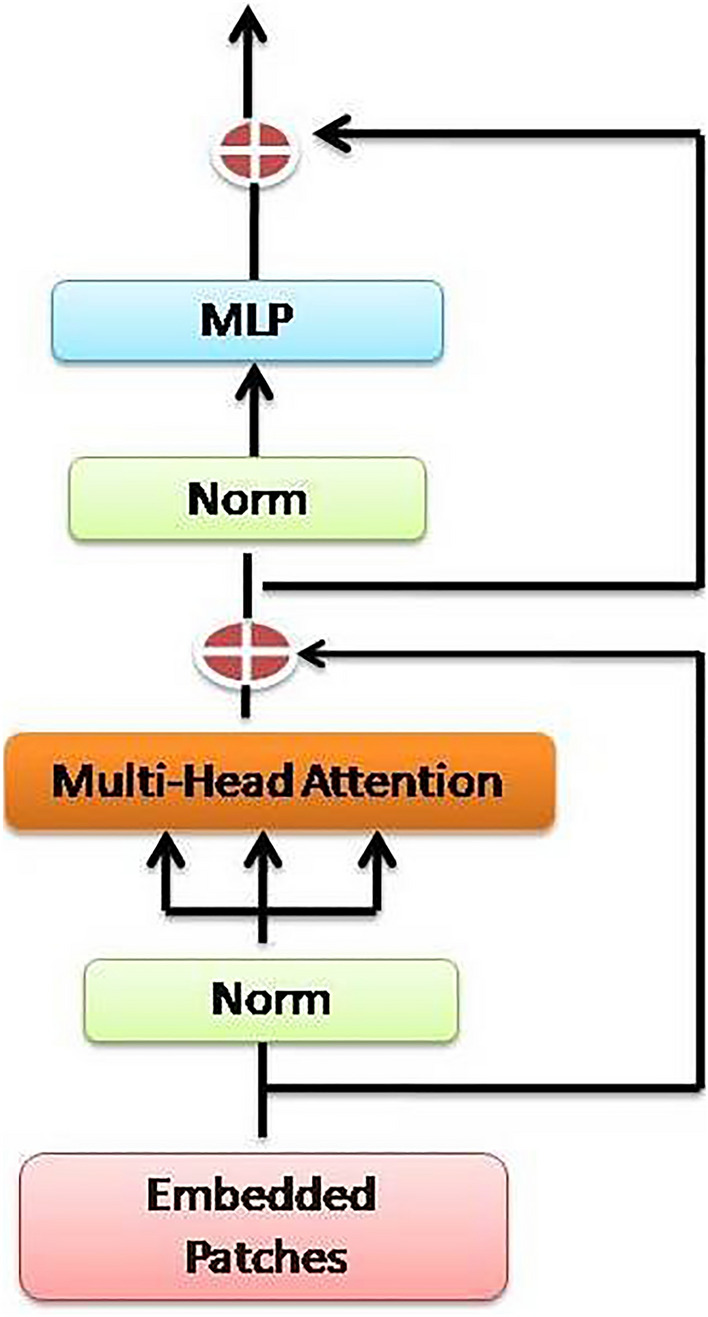
Internal design of a transformer encoder.

Each layer of the Transformer Encoder is constituted of a multi-head self-attention mechanism and a position-wise feed-forward network as shown in Fig. 4.

- Multi-head self-attention: The attention weights between the input embeddings are computed by the multi-head self-attention mechanism. It entails three linear transformations: Query (Q), Key (K), and Value (V), with Q, K, and $V\in R^{(N\times {\text{D}})}$ Using the attention weights, the output of the self-attention mechanism is the weighted sum of the values. The attention weights are calculated by Eq. (1).
  1 $$Attention\left(Q,K,V\right)=softMax\left(\left(Q{K}^{T}\right)/\surd \left(Dh\right)\right)V$$
  where Dh represents the dimension of each attention head.
- Position-wise feed-forward network: The position-wise feed-forward network employs two linear transformations separated by a nonlinear activation function (such as ReLU). Let's designate the attention mechanism's output as $A\in R^{(N\times {\text{D}})}$ The representation of the position-wise feed-forward network is as according to Eq. (2).
  2 $$FFN\left(A\right)={\text{max}}\left(0,A\times W1+b1\right)\times {\text{W}}2+{\text{b}}2$$
  where $$W1\in R^{(D\times {\text{dFFN}})},b1\in R^{(1\times {\text{dFFN}})},W2\in R^{(dFFN\times {\text{D}})},b2\in R^{(1\times {\text{D}})}$$.

These two sub-layers are applied parallel to the input sequence and then combined to generate the encoder layer's output. The process is repeated multiple times to form a stack of encoder layers, where each encoder layer builds upon the representation learned by the preceding encoder layer, enabling the model to learn increasingly complex and generalized representations of the input sequence.

##### Classification layer

This layer utilizes the encoder layers' output to predict pneumonia's presence or absence. This prediction may be made using a fully connected or convolutional layer.

##### Loss function

This component evaluates the model's efficacy based on the predicted and actual labels. In this endeavor, binary cross-entropy loss is a common loss function.

### Ethical standards

No human participants were involved in the study. Dataset is available on Internet.

## Result and discussions

### Performance indicators

Various evaluation metrics are used to measure the effectiveness of machine learning models, and each has its benefits and drawbacks. The most prevalent metrics include.

#### Accuracy

This is the most important metric for evaluating a model and is defined as the proportion of correct predictions to the total number of predictions made by the model. It is evaluated using Eq. (3).

3 $${\text{Accuracy}}=\frac{(\mathrm{True\, Positives }+\mathrm{ True\, Negatives}) }{(\mathrm{True\, Positives }+\mathrm{ True\, Negatives }+\mathrm{ False\, Positives }+\mathrm{ False\, Negatives})}$$

#### Precision

Higher-precision classifiers produce fewer false positives. High accuracy reduces the likelihood of misclassifying negative instances as positive in numerous applications where false positives have severe consequences. Precision is calculated by Eq. (4).

4 $${\text{Precision}}=\frac{\mathrm{True\, Positives}}{(\mathrm{True\, Positives }+\mathrm{ False\, Positive})}$$

#### Recall (sensitivity or true positive rate)

Classifiers with higher recall have fewer false negatives. The classifier captures positive cases and reduces false negatives. A classifier with lower recall has more false negatives. The recall is determined by Eq. (5)

5 $${\text{Recall}}=\frac{\mathrm{True\, Positives}}{(\mathrm{True\, Positives }+\mathrm{ False\, Negatives})}$$

#### F1 Score

The F1 Score is the harmonic mean of precision and recall, indicating patterns between them and calculated using Eq. (6).

6 $${\text{FScore}}=\frac{2 * ({\text{Precision}}*{\text{Recall}})}{({\text{Precision}}\_{\text{Recall}})}$$

#### ROC curve

ROC curves evaluate binary classification models. The model separates positive and negative events across classification thresholds. ROC curve form and position indicate model discrimination. The ROC curve shows the trade-off between positive and negative identification when the classification threshold changes. AUC increases discrimination and model performance.

#### Confusion matrix

The confusion matrix tabulates classification model performance. It compares predicted labels to real labels and shows different classification outcomes. The confusion matrix reveals model performance. True positives (TP) and true negatives (TN) are situations that were accurately predicted. False positives (FP) and false negatives (FN) are cases of misclassification. These values allow us to generate model performance metrics including accuracy, precision, recall, and F1 score.

### Model’s training

To demonstrate our proposed architecture, we experimented with a benchmark dataset of CXR images, one of the most frequently downloaded datasets for testing on Kaggle. Using these studies and datasets for binary classification. Python 3.7, Anaconda/3, and CUDA/10 are installed on a Windows server with an i5 CPU, 2 GB GPU, and 8 GB RAM, as well as an Anaconda/3 distribution. In addition to the parameters listed above, the Python libraries Pytorch, OpenCV, matplotlib, os, math, and NumPy are used. During training, the data is partitioned into batches, and the model's parameters are modified based on each cohort's average loss. The group size dictates the number of samples utilized during each update phase. A larger sample size can speed up the training rate but may require additional memory. CrossEntropyLoss was chosen as the experiment's loss function. During training, the model minimizes this loss function. It computes the negative log-likelihood of expected class probability and actual labels. The training algorithm modifies the parameters of the model. In an experiment, the Adam optimizer was used to alter the learning rate for each parameter based on gradient estimates of the first and second moments. Pytorch was used for the implementation, and training was conducted in a GPU environmentThe learning rate establishes how much model parameters are updated with each optimizer iteration. The multiplicative factor of the learning rate is used to modify the learning rate at each epoch or phase, enabling more granular control of the learning rate during training. The learning rate's multiplicative factor can help the model converge on a superior solution. Table 4 demonstrates the experiment's hyperparameter settings. The novelty of our work lies in the application of the Vision Transformer (ViT), specifically utilizing the DEIT_Base_Patch16_224 pre-trained weights, to the domain of medical imaging for pneumonia detection. While ViT has shown promise in various fields, its adaptation to medical imaging, especially chest X-ray analysis, is relatively unexplored. Our approach capitalizes on ViT's ability to capture intricate spatial relationships in images, offering advantages over traditional methods. We demonstrate improved performance and potential for enhanced pneumonia detection accuracy, marking a significant contribution to the field of medical image analysis.

**Table 4:** Hyper-parameter setting used in the experiment.

Hyperparameter	Value
Batch size	16
Criterion	CrossEntropyLoss
Learning rate	1e − 05
Optimizer	Adam
Device	Cuda
Image resize	224 × 224
The multiplicative factor of the learning rate	0.995

A model's performance depends on these hyperparameters and others. To enhance model performance, selecting hyperparameter values requires careful analysis and experimentation. For optimal performance, hyperparameters must be explored and fine-tuned based on task, dataset, and model architecture.

### Performance evaluation

The model's train-validation accuracy against the epoch curve shows its learning and generalization. If training accuracy increases but validation accuracy plateaus or falls, it indicates overfitting. Convergence and excellent accuracy for both curves show learning and generalization efficacy. The train-validation loss versus epochs curve shows model optimization. The model initially matches data better when training and validation loss decreases. Overfitting occurs when training loss decreases with increasing validation loss. Convergence and low loss suggest error minimization and good generalization for both curves.

**Figure 5:**
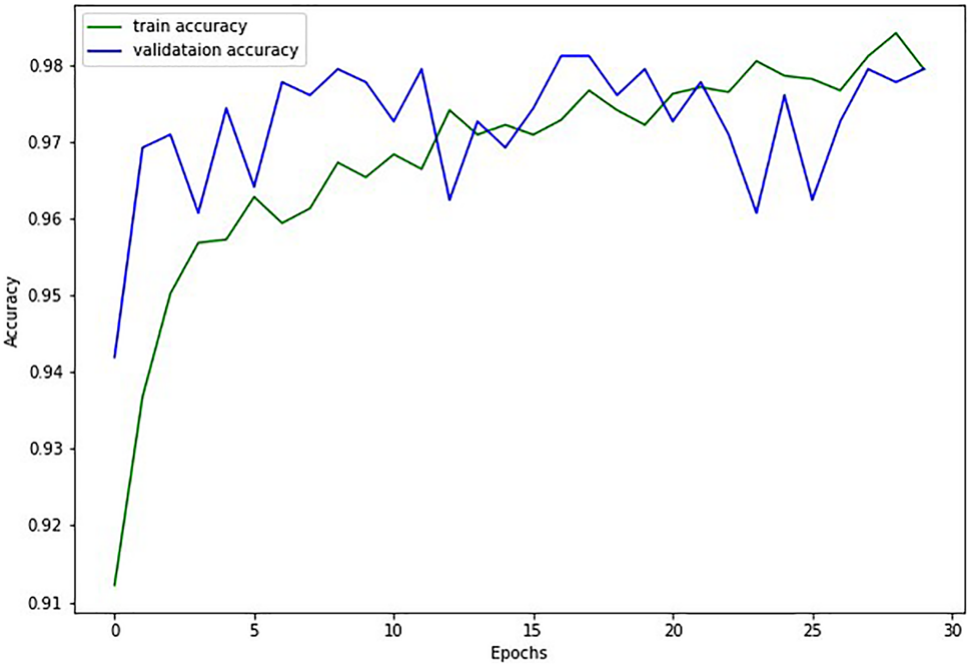
Accuracy variation vs epoch curve.

**Figure 6:**
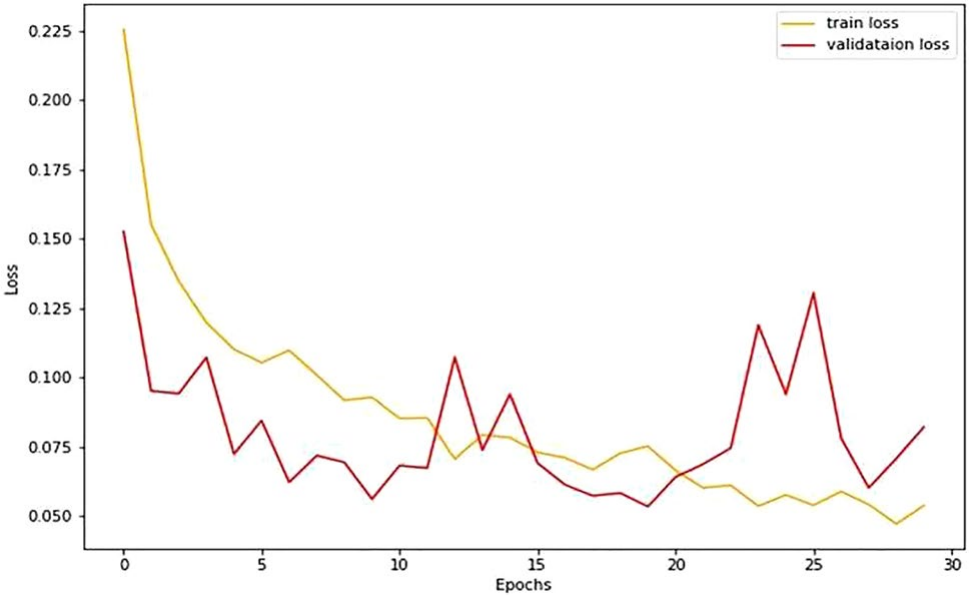
Loss vs epoch curve.

Table 5 presents the performance delivered by the proposed approach and Figs. 5 and 6 show the relationship between accuracy and epoch and loss and epoch, respectively. Figures 5 and 6 show that during training, validation accuracy gradually improves along with test accuracy and reaches 97.61 and other performance indicators are also indicating outperforming results.

**Table 5:** Performance delivered by the proposed model.

Epoch	Split ratio	Loss (train)	Accuracy (train)	Loss (test)	Accuracy (test)	Sensitivity	Specificity	F score	AUC
30	0.20	0.057	98.04	0.069	97.61	0.949	0.981	0.952	0.966

#### Confidence intervals test

This is statistical tool used to estimate the range within which a performance metric, such as accuracy, sensitivity, or specificity, is likely to lie. They provide a range of values that likely contains the true value of the parameter, along with a level of confidence.

Confidence interval (CI) is calculated using the formula described using Eq. (7)

7 $$\mathrm{Accuracy CI}=\mathrm{Accuracy }\pm {\text{Z}}\times \sqrt{\frac{{\text{Accuracy}}\times (1-{\text{Accuracy}})}{\mathrm{sample\, size}}}$$

Z is the z-score corresponding to the desired confidence level. For example, for a 95% confidence level, the Z-score is approximately 1.96.

#### Interpretation

The accuracy reported as 97.61% with a 95% confidence level, the confidence interval is between 96.2 and 98.9%. This means we can be 95% confident that the true accuracy of our proposed model lies within this range.

#### Matthews correlation coefficient (MCC)

The Matthews correlation coefficient (MCC) is a measure used in machine learning to evaluate the quality of binary classification. The formula for MCC is described in Eq. (8).

8 $$MCC=\frac{TP\times TN-FP\times FN}{\sqrt{\left(TP+FP\right)\left(TP+FN\right)\left(TN+FP\right)(TN+FN)}}$$

From the confusion matrix on the test data.

$$\begin{aligned} & {\text{TP}} = {152},{\text{ TN}} = {42}0,{\text{ FP}} = {6},{\text{ FN}} = {8} \\ & {\text{MCC}} \approx 0.{9396} \\ \end{aligned}$$

The Matthews correlation coefficient (MCC) typically ranges from − 1 to + 1:

- + 1 indicates a perfect prediction,
- 0 suggests a random prediction,
− 1 indicates a total disagreement between prediction and observation.

In this case, an MCC of approximately 0.9396 indicates a very strong positive correlation between the predicted and actual classifications. This suggests an excellent classification performance for the model used.

**Figure 7:**
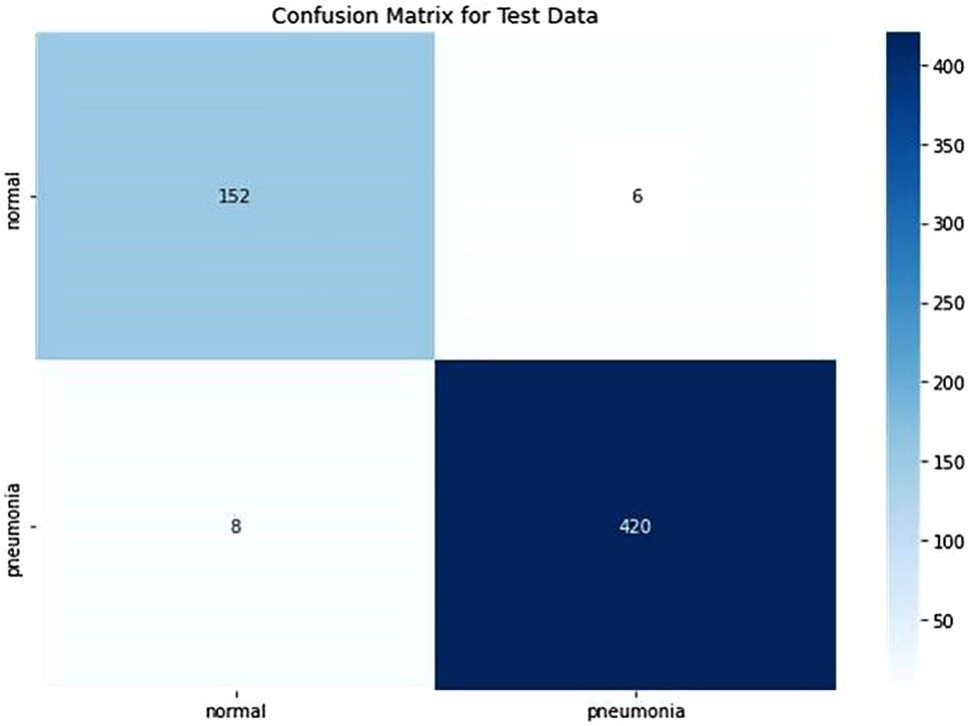
Confusion matrix based on test data for the proposed model.

**Figure 8:**
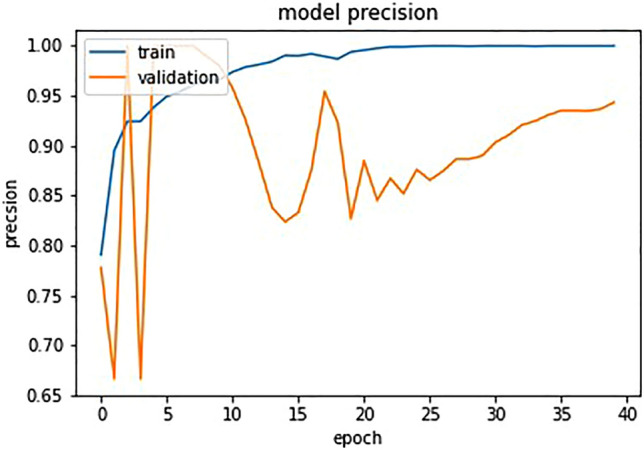
Model precision with epocs.

**Figure 9:**
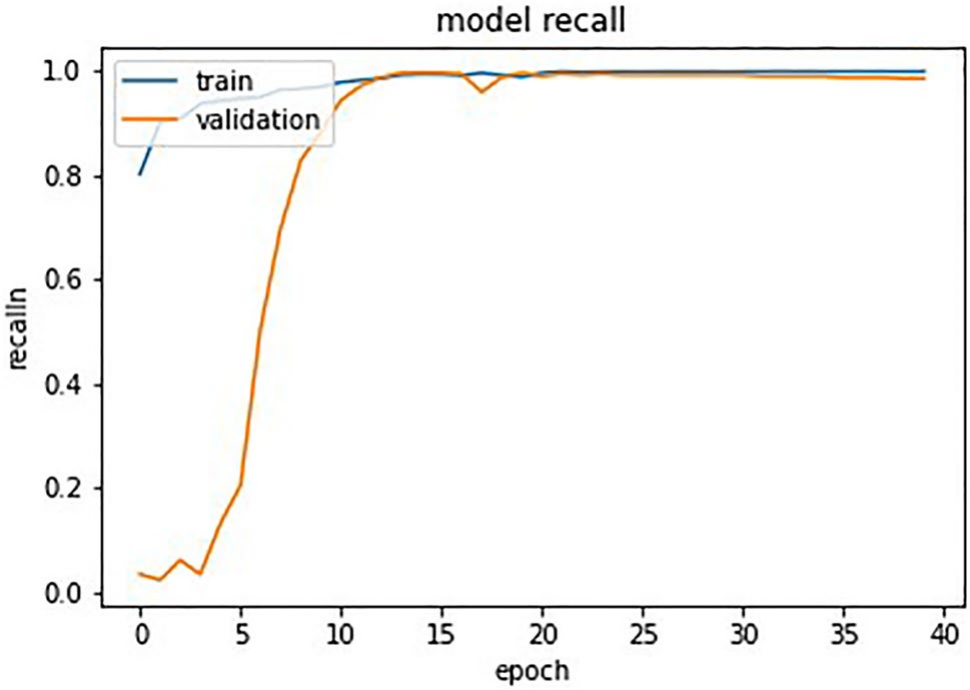
Model recall with epocs.

**Figure 10:**
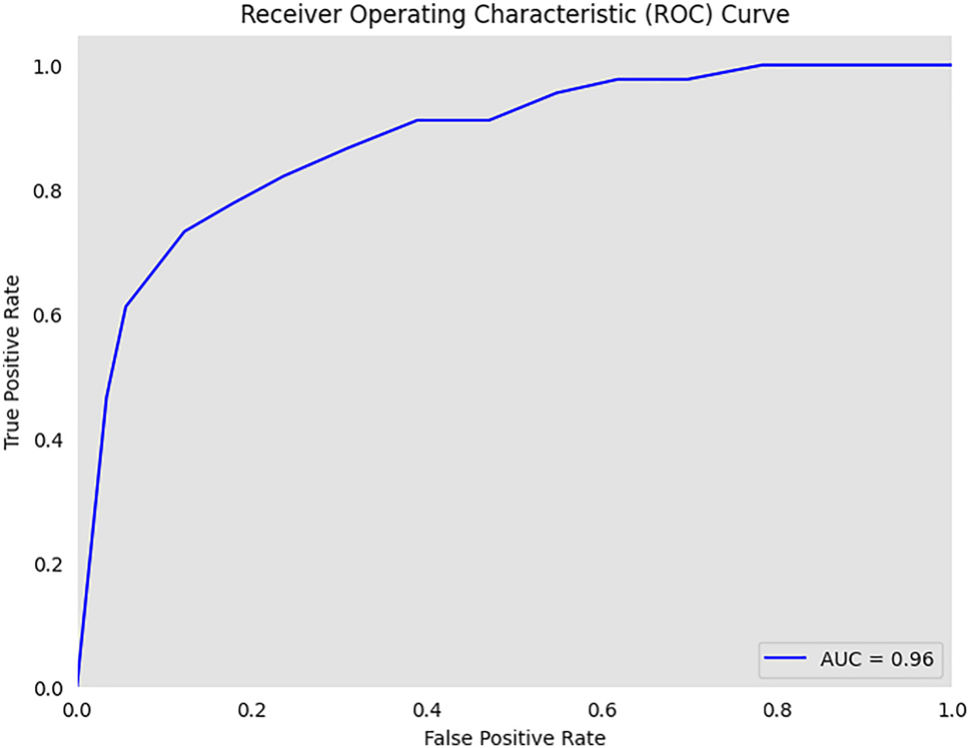
ROC curve with AUC 0.96 of proposed work.

**Figure 11:**
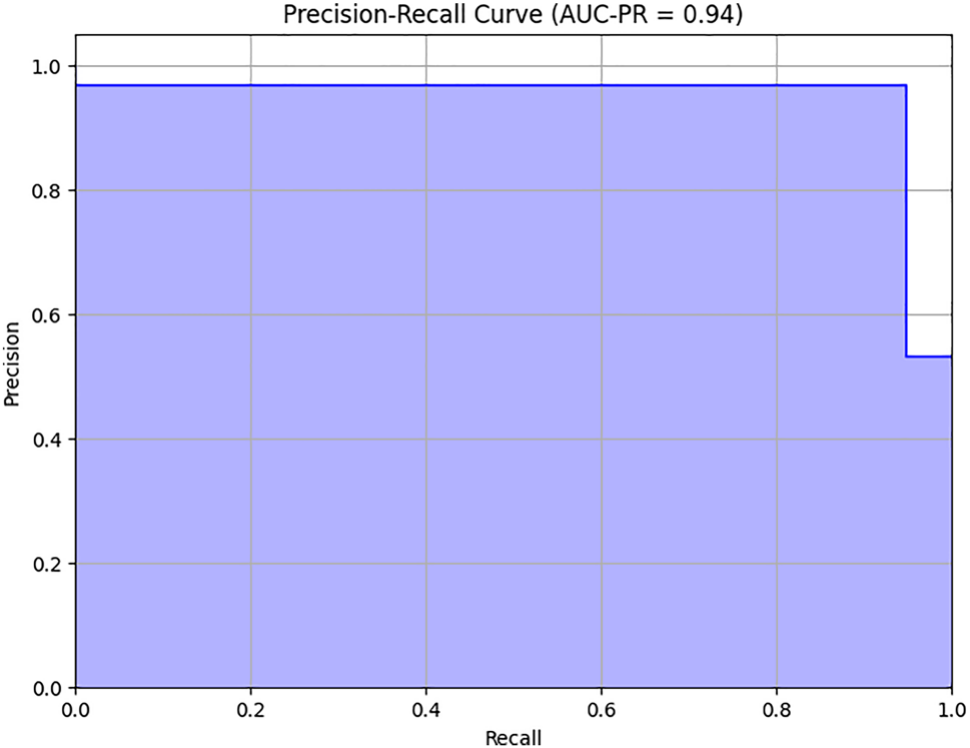
Precision–recall curve of the proposed method.

The confusion matrix in Fig. 7 shows that out of 586 samples in the test data, our proposed model showed 152 cases of TP and 420 cases of TN and 6 cases of FP,and 8 cases of FN which indicates a test accuracy of 97.61%. Variation of precision and recall is represented by Figs. 8 and 9, which indicates that recall converse after 15 epocs while precision converse after 35 epocs. The ROC of the suggested architecture, depicted in Fig. 10, indicates an AUC value of 0.96. It denotes the capability of our proposed model to identify the presence or absence of pneumonia. A precision-recall value of 0.94, depicted in Fig. 11, suggests that the model demonstrates a notable capacity to accurately predict positive instances while capturing a substantial proportion of the true positive instances. The precise interpretation may differ depending on the domain of application and the particular objectives of the classification endeavor.

## Discussion

Table 6 presents the performance of pre-train CNN architectures keeping all hyper-parameters values the same to make a comparison on the same datasets. It shows that Vision Transformer architecture offersa great improvement over all other architectures, The proposed architecture offers an accuracy of 97.61% and an AUC of 0.96 but this more extraordinary performance is obtained by compromising on training time because the training was a bit time taking when compared with different architectures.

**Table 6:** Performance evaluation relative to other architectures utilizing the same dataset.

Sr no.	Architecture	Refs.	Accuracy	F-score	# of trainable parameters	# of non-trainable parameters
1	VGG16	^59^	92.14	0.9234	50,178	14,714,688
2	VGG19	^60^	90.22	0.8999	50,178	20,024,384
3	ResNet50	^61^	82.37	0.8281	200,706	23,587,712
4	ResNet101	^62^	75.96	0.7593	200,706	42,658,176
5	ResNet152	^63^	87.18	0.8734	200,706	58,370,944
6	ResNet50V2	^64^	89.26	0.8937	200,706	23,564,800
7	ResNet101V2	^65^	92.62	0.9250	200,706	42,626,560
8	ResNet152V2	^66^	92.94	0.9312	200,706	58,331,648
9	InceptionV3	^67^	89.42	0.8937	102,402	21,802,784
10	InceptionResNetV2	^68^	90.70	0.8989	200,706	58,331,648
11	DenseNet121	^69^	91.82	0.9171	100,354	7,037,504
12	DenseNet169	^70^	88.78	0.8874	163,074	12,642,880
13	DenseNet201	^71^	91.83	0.9171	188,162	18,321,984
14	NASNetLarge	^72^	88.14	0.8812	975,746	84,916,818
15	Quaternion Residual Network	^73^	93.75	0.9405	560,769	8,576
16	Vision Transformer	Proposed in the paper	97.61	0.9500	85,800,194	0

### Research prospects in Vision Transformer

Vision Transformer (ViT) architecture research prospects for image classification hold tremendous potential for advancing the field. Future research can concentrate on enhancing the performance of ViT models by optimizing their architecture, refining training strategies, and investigating novel techniques to improve precision, robustness, and efficiency. In addition, efforts can be focused on developing interpretability methodologies for ViT models, allowing for a better comprehension of their decision-making process. It is possible to investigate efficient training and inference methods to reduce computational complexity and accelerate model deployment. Adapting ViT to scenarios with limited data using semi-supervised and few-shot learning techniques will increase its applicability. In addition, domain-specific extensions, hybrid architectures that combine ViT with other models, and real-world deployments will contribute to the advancement and practical application of ViT in image classification tasks.

## Conclusion

The article conducts a thorough analysis of a Vision Transformer (ViT) framework for pneumonia detection in chest X-rays. ViTs' ability to analyze complex image relationships is showcased, demonstrating superior performance over traditional CNNs and other advanced techniques. ViTs excel in capturing global context, spatial relations, and handling variable image resolutions, leading to accurate pneumonia detection. The study aims to assess this method's effectiveness by comparing it to state-of-the-art models on a diverse CXR dataset. The results reveal ViT's superiority with an accuracy of 97.61%, sensitivity of 95%, and specificity of 98%. In conclusion, the ViT-based approach holds promise for early pneumonia detection in CXRs, offering substantial development potential in this field. However, limitations include data scarcity and the need for real-world validation. Future directions encompass enhancing interpretability, addressing model robustness, and conducting clinical trials for practical deployment.

## Data Availability

In this work, a public dataset of CXR (https://data.mendeley.com/datasets/rscbjbr9sj/2) has been used.
